# NEUROLOGIC HEALTH: Acrolein and Neuro Disorders

**DOI:** 10.1289/ehp.119-a68a

**Published:** 2011-02

**Authors:** Bob Weinhold

**Affiliations:** **Bob Weinhold**, MA, has covered environmental health issues for numerous outlets since 1996. He is a member of the Society of Environmental Journalists

Neurologic disorders are among the leading causes of death and illness in the United States. Their causes are poorly understood, but one of the emerging suspected culprits is the substance acrolein, which tends to be significantly elevated in the brains or spinal cords of people who have Alzheimer disease, Parkinson disease, amyotrophic lateral sclerosis (ALS), and other neurologic disorders.^1^–^4^ A new study adds multiple sclerosis (MS) to the list of disorders potentially affectedA by this substance.^5^

Acrolein is produced naturally in the body as a by-product of membrane lipid peroxidation. It also occurs in combustion by-products such as vehicle exhaust, industrial emissions, oil- and coal-fired power plant emissions, cooking fumes, and the smoke from burning cigarettes, wood, and plastics. It’s used as a biocide and to manufacture other chemicals and products such as chemical weapons. The U.S. Environmental Protection Agency (EPA) has determined the ubiquitous pollutant is a major source of respiratory damage.^6^ But information on the neurologic effects of environmental acrolein is scant.

In the new study, Riyi Shi of Purdue University and colleagues injected mice with substances known to induce experimental autoimmune encephalomyelitis, an animal model for MS.^5^ Within 2 weeks acrolein–lysine adduct levels in the spinal cord began to rise, peaking at 65% higher than in controls at about day 20. At the same time, the mice began to display significant muscle control problems. Treatment with the acrolein-scavenging substance hydralazine reduced those effects to a great although not significant degree. The researchers also detected significant mitigation of damage to the myelin sheath by hydralazine.

Shi and colleagues say their study provides the first evidence that endogenous acrolein plays a key role in MS. Shi says it’s plausible that environmental acrolein can act in the same general way: “There’s no reason not to believe that the same type of damage could occur.”

Richard LoPachin, a neurochemist and director of research at Montefiore Medical Center in New York, partially agrees. “Because acrolein is highly reactive with proteins at the site of exposure, it has limited distribution in the body and, therefore, limited access to the brain,” he says. But acrolein is just one of many type-2 alkenes, a large family of environmental and food contaminants that includes acrylamide, methyl vinyl ketone, methyl acrylate, and 4-hydroxynonenal. LoPachin says type-2 alkenes share a common mechanism of action at nerve terminals in the brain, and he thinks the combined effects of these substances could contribute to some neurologic disorders.

Robert Kavlock, director of the EPA National Center for Computational Toxicology, says acrolein’s physical properties make it difficult to assess the compound using the agency’s ToxCast™ high-throughput chemical screening program using currently available technology.^7^ But pinning down the causes of these neurologic disorders could help millions of people. In the United States alone, about 5.3 million people have Alzheimer disease,^8^ about 1.5 million have Parkinson disease,^9^ about 400,000 have MS,^10^ and about 30,000 have ALS.^11^
